# Impact of the Timed and Targeted Counselling Model on Maternal Health Continuum of Care Outcomes in Northern Uganda: Protocol of a Quasi-Experimental Study

**DOI:** 10.3390/mps7060098

**Published:** 2024-12-10

**Authors:** Douglas Zibugu, Jessica S. Gubbels, Christabellah Namugenyi, John Bosco Asiimwe, Sanne Gerards

**Affiliations:** 1 Department of Health Promotion, NUTRIM Institute of Nutrition and Translational Research in Metabolism, Maastricht University, 6211 LK Maastricht, The Netherlands; jessica.gubbels@maastrichtuniversity.nl (J.S.G.); sanne.gerards@maastrichtuniversity.nl (S.G.); 2 School of Statistics and Planning, Makerere University, Kampala 7062, Uganda; christabellah.namugenyi@mak.ac.ug (C.N.); john.asiimwe@mak.ac.ug (J.B.A.)

**Keywords:** timed and targeted counselling, community health workers, antenatal care, advised place of delivery, postnatal care

## Abstract

Background: About 287,000 women died globally during their pregnancy journey in 2020, yet most of these deaths could have been prevented. In Uganda, studies show that using Community Health Worker (CHW) visits to households with a pregnant woman can support the prevention of adverse maternal and neonatal outcomes. One such intervention is through the timed and targeted counselling (ttC) approach, where CHWs deliver tailored messages to mothers and their male caregivers at key stages of pregnancy. This study aims to evaluate the impact of the ttC approach on maternal health in Northern Uganda. The main outcomes include antenatal care attendance, advised place of delivery, and postnatal care visit. Methods: We will employ a cross-sectional quasi-experimental design, with retrospective data to compare an intervention group (where ttC is implemented) to a control group (without intervention) using the propensity score matching (PSM) technique applying a 1:1 ratio with a caliper width of 20% of the standard deviation to estimate the average treatment effects. Adjusted odds ratios after generating matched pairs will be reported with 95% confidence intervals with Rosenbaum sensitivity analysis carried out for robustness. Discussion: These findings can be used to modify the implementation of the ttC approach, thereby enhancing its efficiency and effectiveness.

## 1. Introduction

Maternal health refers to the health of women during pregnancy, childbirth, and the postnatal period. Each stage should be a positive experience, ensuring women and their infants reach their full potential for health and well-being [1]. About 287,000 women died during and following pregnancy and childbirth in 2020. Almost 95% of all these maternal deaths occurred in low and lower middle-income countries, and most could have been prevented [2].

Maternal mortality is a global challenge with 223 deaths occurring per 100,000 livebirths worldwide [3]. In Uganda, the maternal mortality rate is 336 deaths per 100,000 live births, which is higher than the UN’s target to reduce the global maternal mortality ratio to less than 70 per 100,000 live births [4,5]. Antenatal care (ANC) refers to the care given to pregnant women by skilled health care professionals (such as health education and promotion professionals) to ensure the best health conditions for both the mother and the baby during pregnancy [6]. This regular contact between the pregnant woman and skilled health care professionals at the health facilities is essential for the health of the women and their children [7].

However, the ANC attendance rate in Uganda is 59.9% for four or more visits [8]. This inadequate utilisation of ANC has been attributed to factors such as long wait times at health facilities, reliance on family or traditional healers for advice, and delayed initiation of care [9]. Compared with home birth, childbirth in a health facility attended by skilled birth attendants is associated with improved maternal and child health outcomes, including lower rates of maternal morbidity and mortality [10,11]. However, in Uganda, only 76.6% of pregnant women deliver in a health facility [12]. The choice for the advised place of delivery (PoD) is often influenced by factors such as social, cultural, and environmental factors [13]. However, regarding Uganda specifically, little is known about factors that influence the use of health facilities for delivery, especially in rural areas [13]. Unfortunately, in Uganda, the knowledge of professionals and policy makers regarding the uptake of early postnatal care (PNC) is also low, hindering the formulation of effective policies aimed at reducing maternal mortality [14]. The first 7 postnatal days account for approximately 65% of maternal deaths, with half (50%) of these deaths occurring within the first postnatal day [15]. However, in Uganda, only approximately 22.5% of new mothers attend at least one PNC visit within six weeks [12].

Studies in Uganda have shown that preventable maternal deaths can be averted by community health worker (CHW) visits to pregnancy households, which also plays a crucial role in improving overall ANC attendance, PoD, and PNC [16,17]. These CHWs use various training packages to effectively deliver messaging to pregnancy households, among which is the timed and targeted counselling (ttC) family-inclusive behavioural change communication approach that targets families expecting a child, especially those most vulnerable and marginalized [18]. The ttC CHWs are part of an already existing structure established by the government of Uganda that links village inhabitants to health units [19]. However, these ttC CHWs receive additional training using the ttC package, which differs from the normal Ugandan government’s training package, as it encourages more timely and targeted visits to pregnancy households with a prescribed message at certain points of the pregnancy [18]. The ttC-trained CHWs are expected to maintain up-to-date ttC registers, detailing records of all the new and existing pregnancy cases, number of visits, situation found at households visited, and to tailor their messages to each individual household’s situation to be more effective [18]. As soon as a pregnancy case occurs and is identified in their routine community visits, they concentrate the ttC intervention on this household by counselling them in order to influence their uptake of life-saving maternal health services such as ANC, PoD, and PNC at the health facilities [18,19]. They apply a dialogue counselling methodology with the pregnant women at the household level, based on the assessment of current needs and practices and the negotiation of progressive improvements. This approach encompasses a wide range of life-saving health practices through appropriately timed messages delivered through interactive storytelling. Importantly, the training package (ttC) seeks to engage both the mother and male caregiver (this should be the male in the household providing support and care to the mother throughout the whole pregnancy journey) as decision-makers, embracing a family-inclusive and gender-transformative model of child health and development in which the positive contribution of male caregivers is emphasized [18]. 

In Northern Uganda, the ttC package is currently implemented by CHWs, based on a facilitator’s manual. To simplify the ttC message, the CHWs use interactive stories to draw different scenarios of problems, best practices and possible solutions faced during and immediately after the pregnancy period. These messages are timed, given at specific intervals and targeted, in order for the message to come neither too early nor too late for the mother, throughout the pregnancy and the postnatal period, to effectively influence the decision making in the households both for the mother and their male caregivers [18].

In recent years, innovative studies have indicated that counselling improved maternal and neonatal health outcomes [20,21]. For example, in Italy, Burgio et al. provided evidence that counselling was effective in managing high-risk pregnancies, and beyond the childbirth period, reduced stress in both the mothers and the fathers [20]. Similarly, in Eastern Uganda, an intervention by CHWs was shown to significantly improve antenatal care attendances [22]. However, according to a WHO study, there is limited evidence-based evaluation knowledge on the impact of the available global training packages for CHWs reviewed including ttC that are designed to improve maternal health continuum of care [23]. This protocol therefore would like to bridge this gap by detailing a study on the impact of the ttC training approach by CHWs on the maternal health continuum of care outcomes in Northern Uganda. Our hypothesis is that ttC will impact the maternal health continuum of care outcomes in Northern Uganda. This hypothesis is based on the available literature that suggests that targeted counselling interventions can improve these outcomes [16,24]. We anticipate that the timely message provided through ttC will encourage women to attend ANC visits [18]. Additionally, the messages raising awareness about the importance of delivering at health facilities are expected to increase the proportion of women delivering at the advised PoD [18]. Furthermore, the ttC visits towards the birth of a child are expected to drive more mothers to go to the PNC visits [18].

### Conceptual Framework

**Figure 1:**
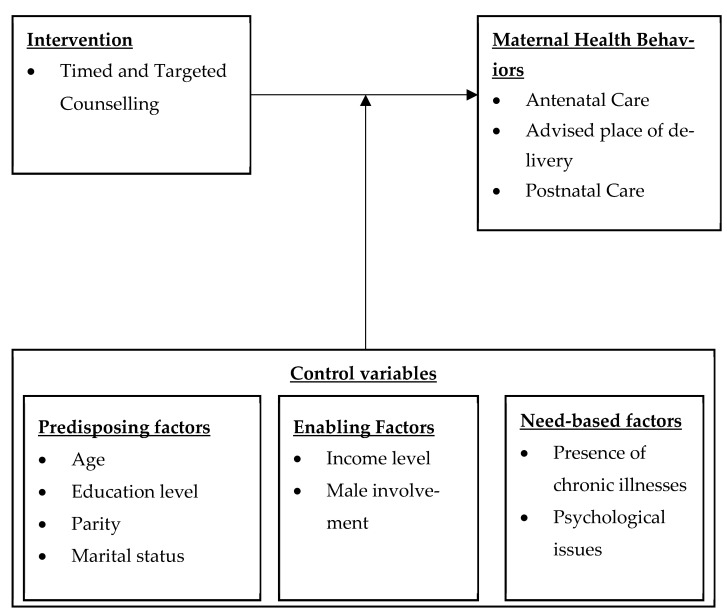
The conceptual framework for studying the impact of ttC on the maternal health continuum of care outcomes, adapted from Andersen’s model [25].

The conceptual framework for this study is based on an adapted Andersen’s Behavioural Model (shown in Figure 1). It shows how maternal health behaviours are influenced by ttC in the presence of various factors proposed by Andersen’s model [25,26]. According to Andersen’s model, predisposing, enabling, and need-based factors affect maternal healthcare utilization [27]. In the current study, predisposing factors include social and demographic factors such as the mother’s age, education level, parity, and marital status. The enabling factors include income level of the household and male involvement in the pregnancy journey. The need-based factors are the presence of chronic illnesses, and psychological issues of the mother. While of importance to the study is the impact of ttC on maternal health behaviours, Andersen’s model helps us to account for how predisposing factors, enabling factors, and need-based factors shape healthcare utilisation. Secondarily, it contributes to understanding the contextual factors influencing maternal health outcomes, which supports efforts to address healthcare access inequality and inform policy development for more equitable access to care [25,26]. 

In this study, we shall incorporate Andersen’s model in investigating the impact of ttC (as an intervention) on ANC utilisation, PoD, and PNC. The ttC is hypothesised to influence maternal healthcare utilisation across the above three aspects by improving mothers’ knowledge on the benefits of fast and sufficient ANC visits, thus improving their attitudes towards ANC. ttC is also hypothesized to improve male-involvement, as well as enhancing the mother’s awareness of their health status and history, thereby affecting PoD and creating need for PNC.

**General Objective**

The main objective of this study will be to assess the impact of ttC on the maternal health continuum of care outcomes in Northern Uganda.

**Specific Objectives**

1. To investigate the impact of ttC on ANC utilisation among pregnant mothers.
2. To evaluate the impact of ttC on the advised place of delivery among pregnant women.
3. To assess the impact of ttC on PNC utilisation among mothers.

**Hypotheses**

The study will be guided by the following hypotheses:

1. ttC implementation will lead to an increase in the utilisation of ANC among pregnant women.
2. ttC will result in a greater proportion of pregnant women delivering at health facilities.
3. ttC will lead to increased PNC utilisation among mothers.

## 2. Methods

### 2.1. Research Design

A cross-sectional quasi-experimental research design with retrospective data collection comparing the intervention group to the control group will be used. Because of the cross-sectional data collection, a randomised control trial is not an option and, therefore, propensity score matching will be used to improve causal inference in this experimental design, as has been used in other similar impact study settings [28,29,30]. PSM will match each treated unit with a nontreated unit of similar characteristics using propensity scores generated from chosen covariates. The preferred characteristics for matching so as to reduce bias and improve precision within the model in this study are education level, birth parity, income, and age of the participants [31]. 

### 2.2. Research Area

The study area will be the northern region of Uganda, specifically the Oyam district, which was selected because ttC is implemented in the Aber subcounty of the district. Northern Uganda in particular is an important region for studying maternal health due to its high poverty rates [32], cultural diversity, limited healthcare access, poor health outcomes, and the presence of conflict-affected individuals [33].

Taking the Oyam district as the sampling frame, two subcounties, Aber and Otwal, are chosen for reaching out to mothers who would have given birth within the past 2–12 months. Aber is the treatment subcounty, and Otwal is the control subcounty because ttC is implemented in the former, but not in the latter. However, the two subcounties of Aber and Otwal were chosen because they are located within the same district and therefore have similar health challenges, such as limited health care access and similar poverty situations in the conflict-affected region [33]. Approximately 18.3% of the population in Aber and 22.3% of the population in Otwal aged 10–19 years have ever been married [34]. Furthermore, in 2021, these two subcounties, among others in Oyam district, had one of the highest teenage pregnancy rates in Uganda [35,36]. Factors mentioned by these new young mothers for the high rate of teenage pregnancy include a lack of knowledge of the ovulation period, inadequate sex education, and social norms that prevent parents from talking to their children about sex education [36]. Significantly, both subcounties are chosen because they do not share borders with each other; thus, limited spillovers are expected from the treatment subcounty to the control subcounty.

### 2.3. ttC Intervention

This ttC package has been identified by WHO as the only available global CHW training package that has supporting material that guide country-specific integration, as opposed to other generic packages available that develop capacities of CHW in maternal health [23]. It targets the first 1000 days (pregnancy and postpartum) of a child’s life [18]. Before implementation of the ttC approach, the CHWs were first trained for 5 days using a standardised ttC package. This training was guided by a developed package that outlined the specific tasks CHWs should, the targeted messages to deliver, and the appropriate timing for these messages [18]. 

After training, the CHWs were then grouped and supervised administratively by district health officials who meet them on a monthly basis for support supervision and review of the ttC household register updates. CHWs then identified households with pregnant women or likely households with reproductive-aged women and encouraged them to register and notify the CHW as soon as a pregnancy case occurred [18].

When a pregnancy is identified by the CHW, they visit these households of the pregnant women in a timely manner during the pregnancy and postnatally, with targeted messages to encourage the pregnant women to attend ANC services and plan to give birth in the presence of skilled personnel and attend to PNC. They further encourage male caregivers to be involved in the whole cycle, from pregnancy through birth to nurturing the baby, by providing both emotional and logistical support. CHWs make four visits during pregnancy before birth and seven visits postnatally in the first 2 years after birth, as explained in Table 1, which summarizes and provides a concise and visually organized reference for the key components and objectives of the ttC approach, as adapted from the ttC manual [18]. 

#### CHW Service Delivery in the Control Subcounty

The CHW in the control subcounty did not follow the ttC approach, but followed the government of Uganda’s national guidelines for recruitment, training, and supervision of CHWs [37]. They were supposed to map out and visit households with pregnant women to ensure that these attended ANC visits and accessed other essential services, but without the timely delivery of targeted messages characteristic of the ttC intervention. They were required to update their CHW registers with progress, which was then reported to the district health officials for supervision. 

### 2.4. Study Population

The study population/sampling frame is composed of respondents at the household level who will be selected from two subcounties, one with mothers who have given birth between 2 and 12 months of age and who participated in the ttC model sessions (with 876 mothers in the treatment group forming part of the sampling frame), or mothers whose access to ttC sessions was limited to the other subcounty of interest (with 1355 mothers in the control group also forming part of the sampling frame). Thus, the total sampling frame/study population will have 2231 mothers from which the sample will be drawn. The mothers will be recruited from the CHW registers that show that they should have attended counselling sessions during their pregnancy. Inclusion criteria are that the mother had a baby between 2 and 12 months. All other mothers are excluded.

### 2.5. Study Instruments

This study will use a cross-sectional primary data source. Structured household questionnaires will be administered to the study participants by the research assistants to gather quantitative data. The questionnaire includes different data types, such as categorical, numerical, and scale options, to facilitate the quantification of information. For validity and reliability, the questionnaire will have embedded skips and rules to ease data collection and perform real-time data cleaning. The study tools/instruments to be used will also include the village health team (CHWs) household registers that register all the mothers at the household level and the ttC register that includes the targeted sessions and number of visits. These will provide the contact and location from which participants will be sampled and the data collected retrospectively.

Tools will be uploaded to Kobo Kollect, a data collection application that will be loaded on phone devices and administered. The Kobo loaded questionnaire will also have mandatory questions marked for emphasis and will have an embedded translation into the local language to ease data collection. A field pretest of the structured questionnaire will be performed before the final roll out.

### 2.6. Data Management

To ensure the integrity and accuracy of the collected data, a robust data management strategy will be implemented. A team of 25 research assistants will be trained and given pre- and post-training tests to test their knowledge with regard to the topic of study and data collection and management processes. The data will be checked for errors at the end of every day, and the research assistant team will meet daily after fieldwork to synchronize their data to a secure server to not only act as a backup, but also to allow the principal investigator to check for validity and accuracy of the tools using the GPS on the Kobo tools loaded on the phones and the time stamps on the tools showing the start and end time for each tool. There will also be ad hoc checks in the field to see that the data are not being manipulated into the system, but are actual data. The data collectors will also be given a unique identifier to enhance the traceability of each tool to individual entrants. Inaccurate information will therefore be easily traced to the research assistant who submitted it to the system, the records will be deleted, and the researcher will stop contributing to the data collection.

### 2.7. Sample Size Calculation

The sample size will be estimated using power analysis from a two-sided Z test, adopted from Charan and Biswas [38], with a 1.5 design effect and a 10% nonresponse rate [39].

This will take into account the desired level of statistical significance, effect size, and expected variability.

The formula is as follows:

$$n=\frac{2(Z_{\alpha /2}+Z_{\beta }{)}^{2}P(1-P)}{(P_{1}-P_{2}{)}^{2}}$$

where:

$Z_{\alpha /2}$ = $Z_{0.05/2}$ = $Z_{0.025}$ = 1.96 is the critical value (from the Z table) at type 1 error, which is the critical value for the chosen level of significance.

$Z_{\beta }$ = $Z_{0.20}$ = 0.842 is the critical value for the chosen power from the standard normal distribution at 80%.

$P_{1}$ = 71.5% is the estimated proportion of the population with the outcome of interest in the control group. This figure is derived by averaging the proportions observed in two previously related studies [24,39] examining one of the outcomes of interest, facility-based deliveries without intervention.

$P_{2}$ = 81.5% is the estimated proportion of the population with the outcome of interest in the intervention group. Like P1, this is also obtained by averaging proportions from the same two related studies [31,32], but in this case, focusing on facility-based delivery with intervention.

P = 76.5% is the pooled prevalence [prevalence in case group ${(P}_{1})$ + prevalence in control group ${(P}_{2})$]/2.

$P_{1}-P_{2}$ = −10% is the difference in the proportion of events in the two groups.

The sample size calculated using the power analysis and taking into consideration the design effect and nonresponse yielded 465 participants in each of the groups (control (465) and treatment (465)) at 80% power and at the 5% level of significance.

### 2.8. Sampling Procedure

To reach the above calculated sample size, a three-stage sampling procedure will be used. First, a simple random sampling within Oyam district will be conducted to select one subcounty where the treatment (ttC implementation) was applied (Aber subcounty) and another where it was not (Otwal subcounty). This random selection ensured that both the intervention and the non-intervention areas were chosen in an unbiased manner.

In the second stage, stratified sampling will be done within the two subcounties to provide the sampling unit, being women of reproductive age (between 18 and 49 years) who have given birth to babies between 2 and 12 months of age. For internal homogeneity within each subcounty, two strata will be formed, one from each subcounty. A total of 930 mothers will be sampled from the 2231 eligible mothers (465 from the 876 available in the intervention subcounty and 465 from the 1355 available in the control subcounty) as per the sample size calculation above. 

**Figure 2:**
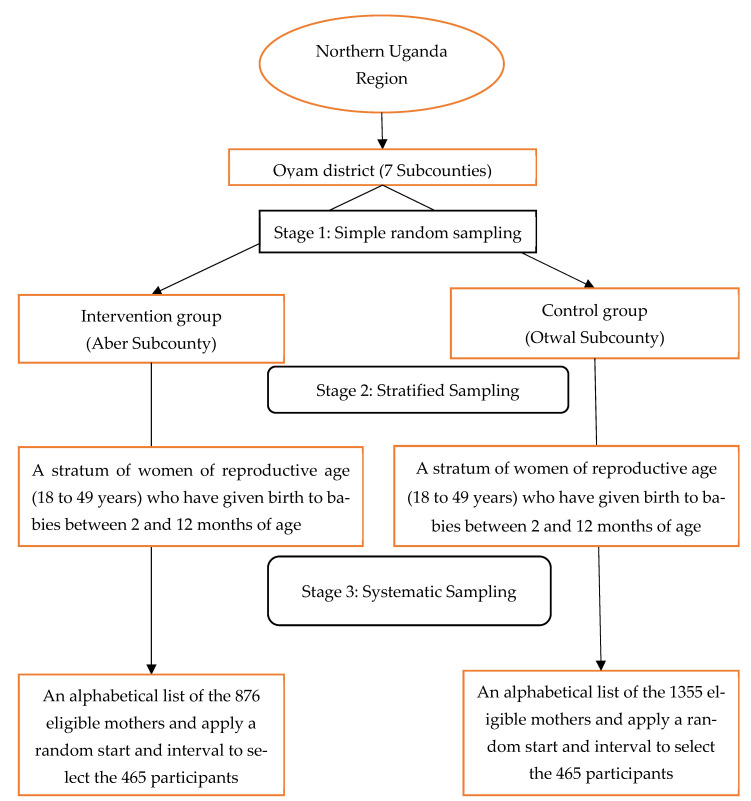
The flow recruitment diagram.

Finally, in the third stage, a systematic sampling method will be applied to randomly select the 930 mothers from an alphabetically compiled list of eligible mothers. A random starting point and a fixed interval will be applied to select participants 465 participants from each subcounty. This process ensures an equal and unbiased representative sample for later generalisation of findings to other similar settings. The flow of this sampling process is shown in Figure 2 below.

By justifying and implementing this comprehensive sampling methodology, the study will aim to enhance the reliability and validity of the research findings.

### 2.9. Study Variables

The primary outcome variables for the study of the impact of ttC on the maternal health continuum of care will be ANC utilisation, skilled birth attendance, and PNC. The ttC variable is nominally defined by whether the household received ttC or not as shown in the Table 2.

### 2.10. Data Analysis

After the data have been cleaned, they will be exported to STATA for analysis and Excel for better visualization. Descriptive and summary statistics will be drawn using the frequencies, measures of central tendency, and dispersion with proportions to provide a descriptive analysis of the context.

There will be cross tabulation of the chosen covariates (education level, birth parity, income, and age) with the intervention (ttC) to obtain their frequency distribution among the treatment and control arms together with the p-values. A logistic regression of the intervention and the covariates will be used to generate the propensity scores. A caliper width at the 20% of the standard deviation will be used for matching participants using the greedy nearest neighbor method in order to get the average treatment effects and the impact of the ttC intervention on the outcomes ANC, PoD, and PNC [40,41].

**Sensitivity Analysis**

Sensitivity analysis will be performed using the Rosenbaum bounds to test the conditional on the unobserved variables, and that the study is free from hidden bias [42]. This will use the rbounds commands in STATA version 14 ranging from 1 to 1.5 with an increment of 0.05. The gamma value will show whether hidden bias is present, as well as the robustness of the results to this potential hidden bias [43]. The sensitivity analysis results for the outcomes will demonstrate whether the treatment effects remain significant and robust to unobserved confounding, even in the presence of hidden bias. This should be across different gamma levels and confidence intervals to ensure the reliability and validity of treatment effects, therefore indicating stability [42,43].

## 3. Discussion

This study protocol describes how the current study will evaluate the impact of the ttC model on the maternal health continuum of care outcomes in Northern Uganda using a cross-sectional quasi-experimental design and employing propensity score matching to control for selection bias [39]. This study will integrate Andersen’s behavioural model, which identifies predisposing, enabling and need-based factors, to obtain a comprehensive understanding of whether emphasizing these factors within the ttC model will result in a more effective maternal health continuum of care outcomes [26]. 

We expect that ttC will significantly improve the maternal health continuum of care outcomes ANC, PoD, and PNC. These outcomes are important to study because they have shown to improve the overall health and wellbeing of both the mother and the child, which reduces the infant and maternal mortality rates globally, thereby contributing to the achievement of Sustainable Development Goal 3 on health and well being [44,45]. Improved ANC utilisation has been linked to early identification of complications, and preventing maternal deaths and comorbid conditions like anaemia [45,46,47]. Ensuring that more women deliver at the advised PoD allows trained professionals to manage both anticipated and unanticipated complications that arise during delivery thereby preventing stillbirths and improving the survival rate of the newborns [48,49]. In addition, since the first days after birth are associated with the highest risks for both the mother and the baby, having PNC visits is essential for the prevention of infection and management of infectious diseases such as HIV and malaria, and maternal anaemia [50,51,52].

This ttC approach, delivered by CHWs to change the maternal care utilisation behaviours of mothers, will likely succeed in the northern Uganda region, which is characterised by inadequate sex education [36], high poverty rates [18], very high teenage pregnancy rates [36], cultural diversity, limited healthcare access, poor health outcomes, and presence of conflict-affected individuals [32]. This is because the ttC approach, as opposed to other available packages, has a distinct advantage of tailoring its message to each household’s characteristics [18,19]. This contextualisation makes the information provided more receptive to the mothers, therefore more relevant and effective as it is relative to their environment, and thus increasing the likelihood of changing behaviours and improving maternal health outcomes. 

Furthermore, adapting Andersen’s behavioural model will give a new perspective on the factors that strongly influence the community, and therefore provide evidence-based insights to emphasize during the modification of the ttC package for use in other conflict-affected regions like northern Uganda [17,18,27].

In conclusion, the evaluation evidence from this impact study is expected to contribute new knowledge on the effects of ttC for similar low-resource and conflict-affected settings [23].

### Strengths and Limitations

This study’s main limitation is the use of a cross-sectional dataset to experiment with an intervention. However, propensity score matching will be used as an alternative to a randomised trial by eliminating the bias due to confounding, and thereby ably applying causal inference for the results [30]. Another limitation would be the several unmeasured confounders which would improve precision if included in the study. This will be mitigated by using sensitivity analysis to sow robustness of the results to these unmeasured confounders [43]. There may be response bias due to self-reporting, which may affect the information gathered. This limitation will be addressed by providing confidentiality assurance to the study participants to allow them to answer more truthfully. Finally, spillover effects will be mitigated by having distant subcounties that do not share borders.

## Figures and Tables

**Table 1:** The overview of key components of the ttC approach.

Timing (Outcome Focused On)	Targeted Counselling Given by CHW
1st visit between 2 and 3 months of pregnancy (ANC)	This should be as soon as possible when the mother misses their period so as to encourage the pregnant woman to go for ANC within the first trimester and identify the home care variability in the household that might be needed by the pregnant woman. The CHWs encourage male caregivers to accompany the women for ANCs. They encourage proper nutrition in pregnancy, uptake of iron/folic acid supplements, one extra meal a day for the pregnant woman, handwashing with soap, and having four or more ANCs.
2nd visit between 4 and 5 months of pregnancy (ANC)	This visit occurs at the middle of the pregnancy and is mainly focused on the disease burden, especially on HIV and AIDS, tuberculosis, and other STI testing and prevention awareness.
3rd visit between 6 and 7 months of pregnancy (ANC, PoD)	This visit is meant to promote delivery at a health facility, assisting the family in drawing up a birth plan, including having skilled personnel available during birth, and talk about available family planning measures to encourage 2-year period child spacing.
4th visit between 8 and 9 months of pregnancy (ANC, PoD)	This is one month before delivery to review the birth plan and reiterate the importance of essential newborn care practices. CHW also encourages exclusive breastfeeding and timely seeking for danger signs.
5th visit during the 1st week of life postnatal (PNC)	CHW encourages essential maternal and neonatal care, access to postnatal and postpartum care, and timely seeking for danger signs.
6th visit at 1 month postnatal (PNC)	Growth monitoring, encouraging vaccination against preventable diseases, and care seeking in case of fever.
7th visit at the 5th month of life (PNC)	CHW encourages complementary feeding and the importance of a balanced diet for the baby between the 6th and 9th months.
8th visit at the 9th month of life (PNC)	CHW encourages breast feeding along with complementary foods between the 9th and 12th months, and encourages uptake of micronutrients such as vitamin A supplements.
10th visit at the 12th month of life (PNC)	Encouraging iron rich foods, vitamin A supplements, deworming, and holistic child development.
11th visit at 18 months of life (PNC)	Giving three to four meals to the child a day, child sleeping under a mosquito bed net.

Note: CHW = community health worker, ANC = antenatal care, HIV = human immunodeficiency virus, AIDS = acquired immunodeficiency syndrome, STIs = sexually transmitted infections, PoD = advised place of delivery, PNC = early postnatal care, ttC = timed and targeted counselling.

**Table 2:** Study variables and their definitions.

Variable	Definition of Variable	Answering Scale
Intervention Variable		
ttC	This is a behavioural change approach implemented through CHWs to pregnancy households with a wide range of life-saving health practices through appropriately timed messages delivered using interactive storytelling to improve maternal health service utilisation. Mothers will be asked whether they received ttC from a CHW during their pregnancy.	1 = Yes 0 = No
Outcome Variables		
ANC	This is defined as visits to skilled health personnel during pregnancy. Mothers will be asked whether they attended any ANC visits during pregnancy.	1 = Yes 0 = No
PoD	This refers to the advised place of delivery, in a health facility. Mothers will be asked where they gave birth, with answering options including private health facilities, government health facilities, traditional birth attendant’s homes, or their own homes.	1 = Yes 0 = No
PNC	This refers to the care given mothers immediately after birth. This includes at least one PNC visit within the first 6 weeks after delivery. Mothers will be asked whether they visited a health facility for PNC after delivery.	1 = Yes 0 = No
Independent Variables		
Predisposing Factors		
Age	Mothers will be asked for their age at their last birthday.	1 = <20 2 = 21–25 3 = 26–30 4 = 31–35 5 = 36–40 6 = >40
Education level	Mothers will be asked for the highest level of school ever attended.	1. Preschool 2. Primary 3. O Level 4. A level 5. Tertiary 6. University 7. Non
Parity	Mothers will be asked how many births they have had in their lifetime, including the most recent one.	
Marital status	Mothers will be asked for their current marital status.	1. Widowed 2. Married or living together 3. Divorced or separated 4. Never married or never living together
Enabling Factors		
Income level	This refers to the income of the household. Mothers will be asked, on average, what is their approximate maximum monthly.	1. <200,000 2. >200,000–<500,000 3. >500,000–<1,000,000 4. >1,000,000–<2,000,000
Male involvement	This is defined by male caregivers actively supporting their families and caring for their women in order to access better health services. The mothers will be asked whether their partner ever accompanied them to any ANC visits.	1 = Yes 0 = No
Need-based factors		
Presence of chronic illnesses	The mothers will be asked whether they have any chronic health conditions or illnesses (such as diabetes, cancer, HIV/AIDS, kidney/heart disease).	1 = Yes 0 = No
Psychological issues	Mothers will be asked whether they experienced any psychological problems during pregnancy (for example anxiety, depression, stress, body image issues).	1 = Yes 0 = No

Note: ANC = antenatal care, PNC = postnatal care, ttC = timed and targeted counselling, CHW = community health worker, PoD = advised place of delivery, HIV/AIDS = human immunodeficiency virus/acquired immunodeficiency syndrome.

## Data Availability

N/A as it is a protocol.
